# Using HIPAA (Health Insurance Portability and Accountability Act)–Compliant Transcription Services for Virtual Psychiatric Interviews: Pilot Comparison Study

**DOI:** 10.2196/48517

**Published:** 2023-10-31

**Authors:** Salman Seyedi, Emily Griner, Lisette Corbin, Zifan Jiang, Kailey Roberts, Luca Iacobelli, Aaron Milloy, Mina Boazak, Ali Bahrami Rad, Ahmed Abbasi, Robert O Cotes, Gari D Clifford

**Affiliations:** 1 Department of Biomedical Informatics Emory University Atlanta, GA United States; 2 Department of Psychiatry and Behavioral Sciences Emory University Atlanta, GA United States; 3 Department of Psychiatry Duke University Health Durham, NC United States; 4 Department of Biomedical Engineering Georgia Institute of Technology Atlanta, GA United States; 5 Department of Epidemiology Emory University Rollins School of Public Health Atlanta, GA United States; 6 Infection Prevention Department Emory Healthcare Atlanta, GA United States; 7 Animo Sano Psychiatry Durham, NC United States; 8 Department of Information Technology, Analytics, and Operations University of Notre Dame Notre Dame, IN United States

**Keywords:** ASR, automatic speech recognition, Health Insurance Portability and Accountability Act, HIPAA, Linguistic Inquiry and Word Count, LIWC, mental health, psychiatric interview, speech to text, WER, word error rate

## Abstract

**Background:**

Automatic speech recognition (ASR) technology is increasingly being used for transcription in clinical contexts. Although there are numerous transcription services using ASR, few studies have compared the word error rate (WER) between different transcription services among different diagnostic groups in a mental health setting. There has also been little research into the types of words ASR transcriptions mistakenly generate or omit.

**Objective:**

This study compared the WER of 3 ASR transcription services (Amazon Transcribe [Amazon.com, Inc], Zoom-Otter AI [Zoom Video Communications, Inc], and Whisper [OpenAI Inc]) in interviews across 2 different clinical categories (controls and participants experiencing a variety of mental health conditions). These ASR transcription services were also compared with a commercial human transcription service, Rev (Rev.Com, Inc). Words that were either included or excluded by the error in the transcripts were systematically analyzed by their Linguistic Inquiry and Word Count categories.

**Methods:**

Participants completed a 1-time research psychiatric interview, which was recorded on a secure server. Transcriptions created by the research team were used as the gold standard from which WER was calculated. The interviewees were categorized into either the control group (n=18) or the mental health condition group (n=47) using the Mini-International Neuropsychiatric Interview. The total sample included 65 participants. Brunner-Munzel tests were used for comparing independent sets, such as the diagnostic groupings, and Wilcoxon signed rank tests were used for correlated samples when comparing the total sample between different transcription services.

**Results:**

There were significant differences between each ASR transcription service’s WER (*P*<.001). Amazon Transcribe’s output exhibited significantly lower WERs compared with the Zoom-Otter AI’s and Whisper’s ASR. ASR performances did not significantly differ across the 2 different clinical categories within each service (*P*>.05). A comparison between the human transcription service output from Rev and the best-performing ASR (Amazon Transcribe) demonstrated a significant difference (*P*<.001), with Rev having a slightly lower median WER (7.6%, IQR 5.4%-11.35 vs 8.9%, IQR 6.9%-11.6%). Heat maps and spider plots were used to visualize the most common errors in Linguistic Inquiry and Word Count categories, which were found to be within 3 overarching categories: Conversation, Cognition, and Function.

**Conclusions:**

Overall, consistent with previous literature, our results suggest that the WER between manual and automated transcription services may be narrowing as ASR services advance. These advances, coupled with decreased cost and time in receiving transcriptions, may make ASR transcriptions a more viable option within health care settings. However, more research is required to determine if errors in specific types of words impact the analysis and usability of these transcriptions, particularly for specific applications and in a variety of populations in terms of clinical diagnosis, literacy level, accent, and cultural origin.

## Introduction

In 2020, 52*.*9 million (21%) of US adults experienced a mental illness, and of those, 41*.*4 million (17%) received mental health services either through inpatient treatment, outpatient treatment, or prescription medication [1]. Workforce shortages, specifically within mental health, have been well-documented and are projected to be a persistent concern in the future [2-4]. There is expected to be an insufficient supply of mental health practitioners to meet the need for psychiatric care by 2030, with the number of psychiatrists decreasing by 20% [2]. This shortage is even more pronounced in certain geographic areas due to an uneven distribution of psychiatrists and other mental health practitioners, further limiting access to care [2,4]. To assist with these shortages, many have proposed increased use of telehealth and other technology-assisted solutions to increase efficiency. One promising approach is to use automatic speech recognition (ASR) systems to convert speech into readable text or transcriptions.

The evolution of ASR systems over the years has been marked by a significant reduction in the word error rate (WER), a key metric in evaluating these systems. This is consistent with the decline observed in the WER across the literature. From approximately 30% in the early 2000s [5] to 10% to 15% in the 2010s [6], and subsequently dropping below 10% in recent years [7], the WER trend reflects this evolution. In recent years, the WER of ASR systems applied to the data set (Librispeech Other) demonstrates this trend: Panayotov et al [8] recorded 13.97%, Zeghidour et al [9] achieved 11.24%, Irie et al [10] attained 10.3%, and Whisper (OpenAI Inc) [11] impressively reached a WER of 5.2% [11]. Further insights from diverse data sets and models can be found elsewhere ([11] or Park et al [12]).

ASR has been explored in various clinical applications and continues to grow in popularity. One of the most notable uses of ASR in a clinical setting is to assist the practitioner with clinical documentation in the electronic health record (EHR) [13,14]. EHRs have been cited as a contributing factor to physician burnout due to the significant increase in time spent completing documentation, which has decreased time spent with patients [15,16]. Recommendations to alleviate these challenges have included improving EHRs through ASR technology [17]. Automated medical scribe services may decrease administrative burden and lessen physician burnout related to documentation [13]. Another application of ASR is to assist with clinical education. For example, automated transcriptions can be used in psychotherapy supervision contexts by reducing the time spent generating human transcriptions, providing more timely feedback, and quantifying other relevant information, such as the amount of time spent talking by both the therapist and the client [18]. Research has also begun to use ASR in predictive models to assess, diagnose, and track mental illness [19-21].

One of the most commonly used natural language tools in text analysis is the Linguistic Inquiry and Word Count (LIWC) [22]. The most up-to-date version, LIWC-22, has an internal dictionary of over 12,000 words categorized into various groups intended to assess different psychosocial constructs [22]. Numerous categories related to first-person pronoun use and negatively valenced emotion and tone words have been shown to be associated with depression symptom severity [23-25]. Similarly, people experiencing psychosis use more personal pronouns, negative emotion words, biological process words, and fewer words per sentence compared with controls [26,27].

Although there is a growing interest in ASR applications in clinical practice, few studies have compared the accuracy of commercially available regulatory-compliant, for example, the Health Insurance Portability and Accountability Act (HIPAA) of 1996 [28], transcription services in mental health settings [29]. HIPAA-compliant services provide a regulatory standard for protecting an individual’s identity. Previous research that looked at ASR services’ WERs used Google Cloud speech-to-text after going through an 8-month process to obtain a waiver from their university and having an existing business associate agreement with Google [29]. This process will not be reasonable for many settings; therefore, this study seeks to compare HIPAA-compliant services such as Amazon Transcribe (Amazon.com, Inc) and Zoom-Otter AI (Zoom Video Communications, Inc), along with the latest state-of-the-art open-source software, Whisper, implemented on a local HIPAA-compliant server on which the study data was located. Furthermore, this study also compares the WERs of these transcription services by analyzing groups with different clinical diagnoses. Although ASR services are constantly improving, there is a need to continue to compare these services for a variety of populations. This study compares the WER of multiple commercially available ASR services against human transcriptions using clinical interviews from controls and those experiencing various mental illnesses. The eventual aim of assessing ASR services is to develop a scalable, timely, and cost-effective system for automatically analyzing the semantic content of telemedicine visits to assist in diagnosis and treatment recommendations.

## Methods

### Overview

**Figure 1:**
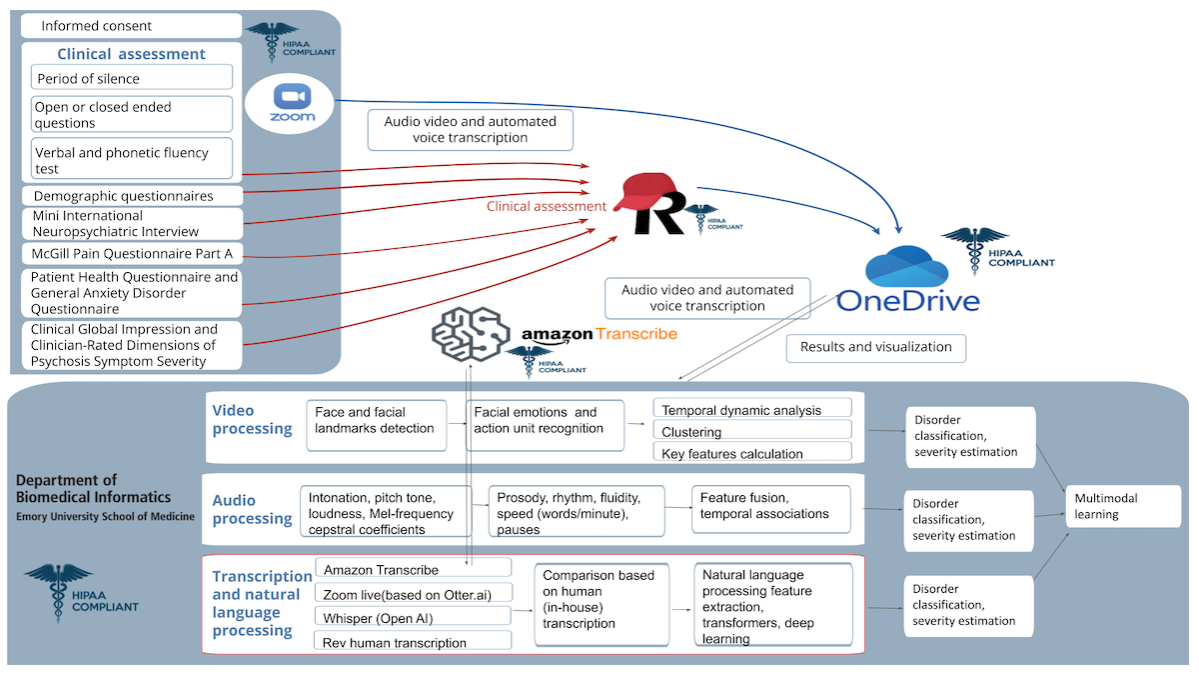
Schematic flow of data collection, storage, and processing. The process of administering the interviews is listed on the left. The clinical categorizations then flow into REDCap (Research Electronic Data Capture; Vanderbilt University; red lines). Audio and video recordings are captured during the interview and placed in the OneDrive (Microsoft Corporation) folder. In this work, we focus on the part of the project shown in the lower red box. Otter AI transcriptions are produced afterward. Data are then synchronized to local servers, which process the same audio data to shorten the length of the files and then transcribe with OpenAI’s Whisper software, Amazon Transcribe, and Rev transcription service (human). Further audio and video processing will be implemented when the transcription process has been fully validated. Adapted from Cotes et al (CC BY [Creative Commons Attribution license] open-source license).

The overall project protocol can be found in Cotes et al [30]. In Figure 1, we have detailed only the parts of the project pertinent to evaluating the automatic and human transcriptions used in the study, highlighted in red.

### Recruitment

Interviewees were recruited from Research Match [31], a National Institutes of Health–funded, web-based recruitment strategy designed to connect potential participants to research studies, and through Grady’s Behavioral Health Outpatient Clinic using a database of interested research participants. Participants were aged between 18 and 65 years and were native English speakers. All interviewees were in the United States at the time of the interview. For the initial screening, interviewees were recruited for either a control group (no history of mental illness within the past 12 months) or a group currently experiencing depression. All diagnoses and group categorizations were verified and finalized by the overseeing psychiatrist and clinical team after the semistructured interview.

### Interviews

All interviews were conducted remotely through Zoom’s (Zoom Video Communications) secure, encrypted, and HIPAA-compliant platform. The interview guide and protocol were created by the study team with components that simulate a psychiatric intake interview [30]. The interview was divided into three parts: (1) a semistructured interview composed of a series of open-ended questions, a thematic apperception test (TAT) [32], a phonetic fluency test [33], and a semantic fluency test [34]; (2) a sociodemographic section; and (3) clinical assessments, which included the Mini-International Neuropsychiatric Interview (MINI) 6.0 [35], McGill Quality of Life Questionnaire [36], General Anxiety Disorder-7 [37], and Patient Health Questionnaire-9 [38]. The upper left side box in Figure 1 contains the visual representation of this flow.

### Categorization

The final sample included 65 interviewees that were categorized into 1 of 2 groups: control (n=18) or mental health condition (MHC) (n=47). Over half (14/18, 78%) of the individuals in the control group did not code into any current or past major depressive disorder, and those with a past history of major depressive disorder (4/18, 22%) all confirmed that their last 2-week episode of depression did not occur within the prior previous 12 months. Individuals with a mental health condition (MHC) had either a primary diagnosis of major depressive disorder (35/47, 75%), some sort of psychosis or manic disorder (9/47, 19%), or a primary anxiety or obsessive-compulsive disorder (3/47, 6%). These individuals also experienced comorbidities such as agoraphobia (18/47, 38%), generalized anxiety disorder (14/47, 30%), posttraumatic stress disorder (4/47, 9%), social anxiety disorder (6/47, 13%), or substance abuse or dependence disorders (5/47, 11%). All classifications were discussed and verified by the overseeing psychiatrist and research team. Interviewees who were unable to participate in the interview or who had a history of traumatic brain or neck injury were excluded. All interviewee demographics are shown in Table 1.

**Table 1:** Interviewee demographics for each interviewee group.

Demographics		Control group (n=18)	MHC^a^ group (n=47)	All participants (n=65)	*P* values	
**Age (y)**						.03^b^
	Mean (SD)	46 (14.19)	38 (13.98)	40 (14.40)		
	Median (IQR)	48 (33.25-59.00)	33 (26-49.5)	36 (26-52)		
**Gender, n (%)**					.10^c^	
	Female	10	35	45		
	Male	8	9	17		
	Nonbinary	0	3	3		
**Race, n (%)**						.19^c^
	Asian	1	6	7		
	Black or African American	7	7	14		
	Hispanic or Latino	0	3	3		
	White	9	30	39		
	Mixed race	1	1	2		
**Years of education**						.78^b^
	Mean (SD)	17 (5.20)	17 (2.52)	17 (3.45)		
	Median (IQR)	16 (14.50-18.75)	17 (15-18)	17 (15-18)		
**Education Level, n (%)**						.27^c^
	Some high school	1	1	2		
	High school graduate	0	2	2		
	Some college, trade, or vocational school	4	11	15		
	College graduate	6	6	12		
	Graduate or professional school	7	27	34		

^a^MHC: mental health condition.

^b^Mann-Whitney Test.

^c^Fischer exact test.

### Automatic Transcription Process

The automatic transcription services used in this study were: Amazon Transcribe, Zoom live transcription (using OtterAI), and Whisper (an open-source ASR system by OpenAI). Amazon Transcribe and Zoom-Otter AI have HIPAA-compliant services that were used in this study. The Whisper ASR was downloaded and used on the local HIPAA-compliant servers. Zoom allows for recording separate audio tracks; therefore, the interviewees’ audio files were used to produce all automatic transcripts. The interviewee-sided audio was played as input within a recorded Zoom meeting (muted with shared audio) with live transcription to produce a text file for only the interviewee-sided audio. To reduce costs, interviewee audio files were edited to remove silences longer than 1 second from the files based on an average amplitude threshold of 5 in 1 second. These shortened audio files showed lower errors and were used as input to create Amazon Transcribe and Whisper transcripts.

### Human Transcription Process

Gold standard transcriptions were produced by the research team using a multiple-overread and consensus approach and were used throughout this study as the gold standard.

We followed the earlier work of Neamatullah et al [39] for the deidentification of medical data. Given that algorithms are sensitive but not specific and humans are the opposite, by combining the strengths of both and adding a human overread step, they demonstrated that this was a highly effective process that neither distorted medical data nor leaked protected health information. In this study, the automated transcriptions for Amazon Transcribe and Zoom-Otter AI were used to produce a side-by-side comparison text document to serve as the basis for human overreads. A total of 2 clinically trained experts overread the entire transcription while listening to the audio. Where the 2 transcriptions disagreed, the computer code highlighted the section with an underscore to help draw the human’s attention to the issue. The research team then edited a separate file to create a correct overread, or gold standard. All numerical quantities were transposed to their word equivalents, slang was written phonetically, and brackets were used to denote utterances such as laughter. Any discrepancies in the transcripts were resolved by the consensus of the 3 clinical transcribers.

The research team also created interviewee-sided transcriptions using a commercial human transcription service offered by Rev. These transcriptions were generated using the shortened audio files previously used to create the Amazon Transcribe and Whisper automated transcriptions. These transcriptions were not used in the process of creating the gold standard but, rather, were generated as a baseline for human performance.

### WER Process

#### Preprocessing and Text Preparation

Each transcript was preprocessed by expanding contractions, removing annotations, and changing all characters to lowercase. Different notations for different transcripts were recognized, and labels and descriptions were removed (for instance, “[Laughter]”). Also, a specific dictionary was built to alleviate error counting between transcriptions based on stylistic preferences (eg, “twenty twenty two,” “two thousand twenty-two,” and “two thousand and twenty-two” were all replaced with “2022”).

#### WER Calculation

**Figure 2:**
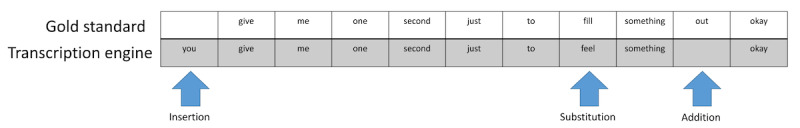
Illustration of the word error rate (WER) calculation. The word “you” is inserted by the transcription engine but has not been spoken. The word “fill” is substituted by “feel” by the transcription engine. Both the words “fill” and “feel” are counted as substitutions, but “fill” would be counted as a substitution deletion (S-delete), and “feel” would be counted as a substitution insertion (S-insert) within this study. The word “out” is deleted by the transcription engine, although it has been spoken.

The gold standard transcriptions produced by interviewers were used as the reference, and all other transcriptions’ performances were compared with the gold standard using WER, a common and important metric for measuring the accuracy of transcriptions [40,41]. The WER, expressed as a percentage, is defined as:



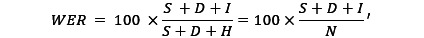



where *S* is the number of substitutions, *D* is the number of deletions (words spoken but left out in the transcription, such as the word “out” in Figure 2), *I* is the number of insertions (words that are not spoken but have been inserted by the transcription, such as the word “you” in Figure 2), *H* is the number of hits (correct words), and *N* is the total number of words in the reference (gold standard). The *Jiwer* library [42] was used to calculate the WER. The library is based on the minimum-edit distance calculated using the Levenshtein distance [43].

The substitution words that are counted in *S* can be divided into 2 parts. The *S*-deletes are the words that are being substituted (“fill” in Figure 2), and the *S*-inserts are substitution words (“feel” in Figure 2). We bundle the error words that are counted in insert (*I*) and those that are in *S*-insert in one category called “Error Insert.” Then the other half, which are words in *S*-delete and words counted in *D* together, are called “Error Delete.”

### Statistical Tests

All *P* values were calculated for 2 sets at a time. A Wilcoxon signed rank test (a nonparametric test) was used for correlated samples, such as when comparing the WER for each interview between different transcription services. The Brunner-Munzel test was used for the independent sets, for example, when comparing the WER between genders or between groups of individuals with different clinical diagnoses. The statistics package *SciPy* (version 1.7.3; Python Library) was used for the calculations.

### Ethical Considerations

The Emory University Institutional Review Board and the Grady Research Oversight Committee granted approval for this study (IRB #00105142). All participants provided informed consent to partake in the study, and all collected data were deidentified by providing a unique identifier to each participant. All participants were compensated for their time volunteering in this study and were provided a 1-time financial incentive (US $30).

## Results

Overall, the interviews lasted 46 minutes on average, with the shortest interview lasting 25 minutes and the longest lasting 1 hour and 55 minutes. Before comparing the clinical groups, WER across genders was compared for each of the 4 services. We did not see any statistical difference for WER between male and female individuals for Amazon Transcribe (*P*=.71), Zoom-Otter AI (*P*=.39), Whisper (*P*=.79), or Rev (*P*=.42). The number of interviewees identifying as nonbinary was too low for any statistical analysis. The comparison of WER for race between individuals who identify as White versus those who did not identify as White did not show any statistically significant difference for Amazon Transcribe (*P*=.17), Zoom-Otter AI (*P*=.26), Whisper (*P*=.25), or Rev (*P*=.13). The groups of individuals who did not identify as White had counts that were too low for any statistical analysis, and thus, we turned this into a binary analysis.

The median of total words spoken by each interviewee was 1280 (IQR 927.0-2041.0). The median word counts for the control and MHC groups were 1337 (IQR 802.5-1961.5) and 1232 (IQR 969.0-1964.0), respectively. Transcriptions were generated using Whisper for both medium and large models. The medium model was chosen due to its superior performance.

### Comparing Clinical Categorizations by Transcription Service

**Figure 3:**
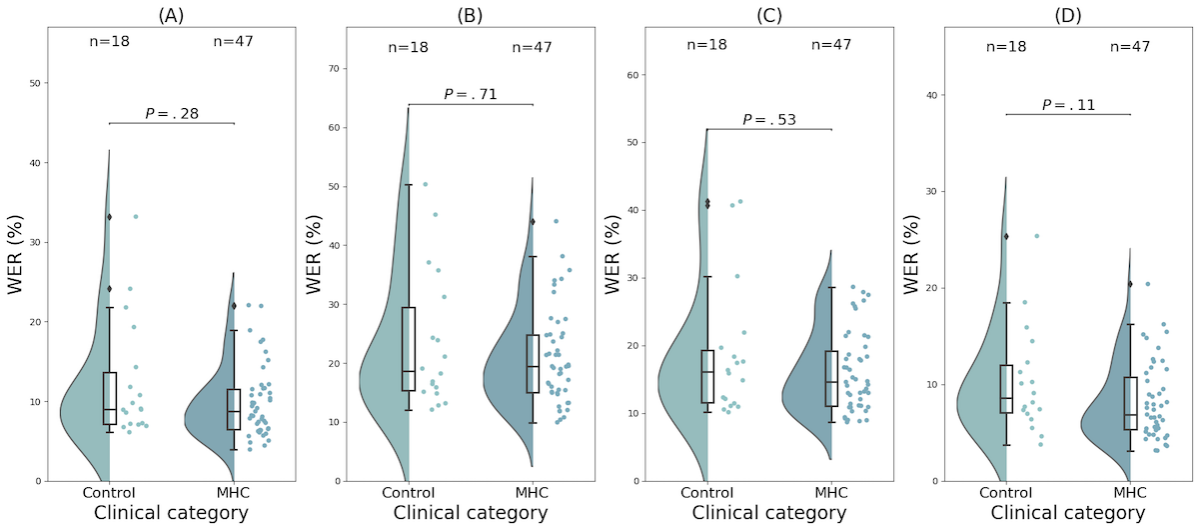
Distributions of the word error rates (WERs) for the control group and the mental health condition (MHC) group within (A) Amazon Transcribe, (B) Zoom-Otter AI, (C) Whisper, and (D) Rev human transcription. Distributions are estimated from actual values (dots) using a kernel density estimate. Box plots indicate the median with the 25th and 75th percentiles. Note that with (*P*>.05) for every Brunner-Munzel test applied between 2 categories, there were no statistically significant differences in WER between the control (n=18) and MHC (n=47) groups within each transcription method.

The median WER values for the control group were lowest for Rev transcription at 8.6% (IQR 7.0%-12.0%), followed by Amazon Transcribe at 9% (IQR 7.2%-13.7%), Whisper at 16.1% (11.6%-19.3%), and Zoom-Otter AI at 18.6% (IQR 15.3%-29.5%). This same trend was seen for the MHC group with Rev transcription’s WER value being 6.9% (IQR 5.0%-10.8%), followed by Amazon Transcribe’s WER value of 8.7% (IQR 6.4%-11.6%), Whisper’s WER value of 16.1% (IQR 11.1%-19.2%), and Zoom-Otter AI’s WER value of 19.4% (IQR 15.0%-24.8%). Using the Brunner-Munzel statistical test comparing each group in a pair-wise manner, there was no statistically significant difference between the clinical groups’ WER for any of the transcription services (all *P*>.05). The breakdown of the WER for transcripts based on the 2 clinical groupings and related *P* values can be seen in Figure 3. Since there were no significant differences found between the clinical groups for each transcription service, the total sample (N=65) was used to compare transcription services with one another for the remainder of all analyses.

### Comparing Automatic Transcription Services

**Figure 4:**
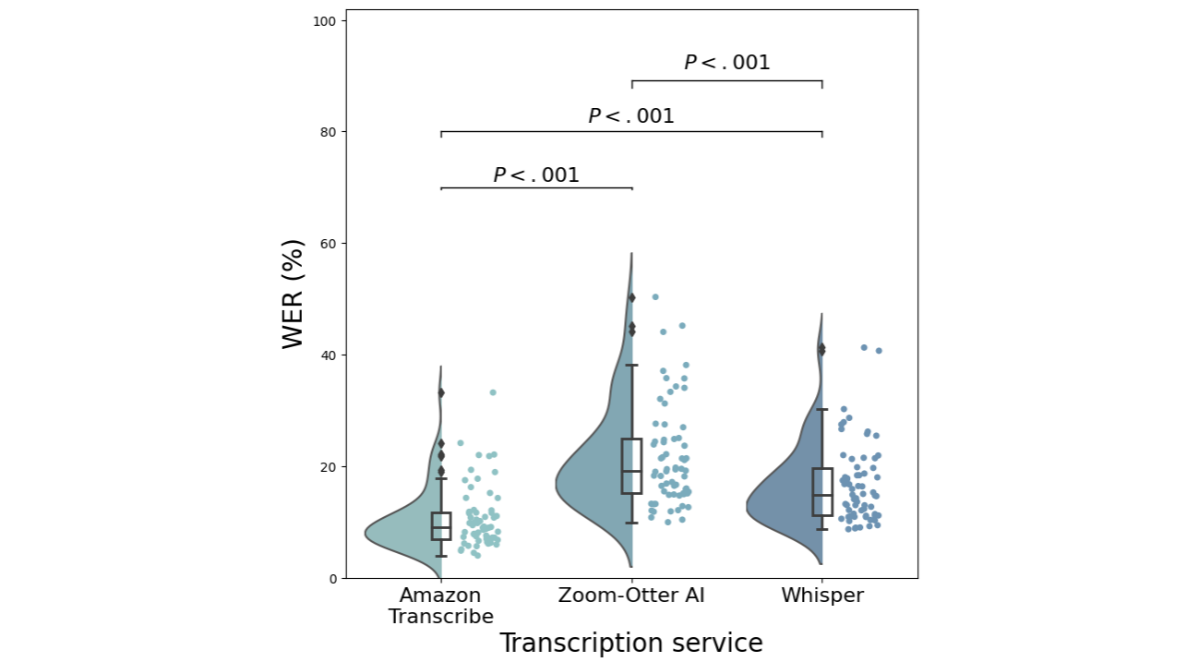
Distributions of the word error rates (WER) for Amazon Transcribe, Zoom-Otter AI, and Whisper transcriptions. Dots indicate the actual values of each WER for each of the (N=65) interviewees. Distributions are estimated from these values using a kernel density estimate. Box plots indicate the median with the 25th and 75th percentiles. *P* values are for a Wilcoxon signed rank test applied between distributions in a pair-wise manner.

The median WER of the 3 tested automatic transcription services was lowest in the Amazon Transcribe transcriptions at 8.9% (IQR 6.9%-11.6%); followed by Whisper at 14.8% (IQR 11.1%-19.7%); and lastly, Zoom-Otter AI at 19.2% (IQR 15.1-24.8). A Wilcoxon signed rank test applied to the WER showed a statistically significant difference between Amazon Transcribe and Zoom-Otter AI (*P*<.001), Amazon Transcribe and Whisper (*P*<.001), and Zoom-Otter AI and Whisper (*P*<.001). Figure 4 provides the distributions of the WER for each service. Amazon Transcribe had the lowest WER of all automatic transcription services and was then compared with the paid human transcription provided by Rev.

### Rev Human Transcription

**Figure 5:**
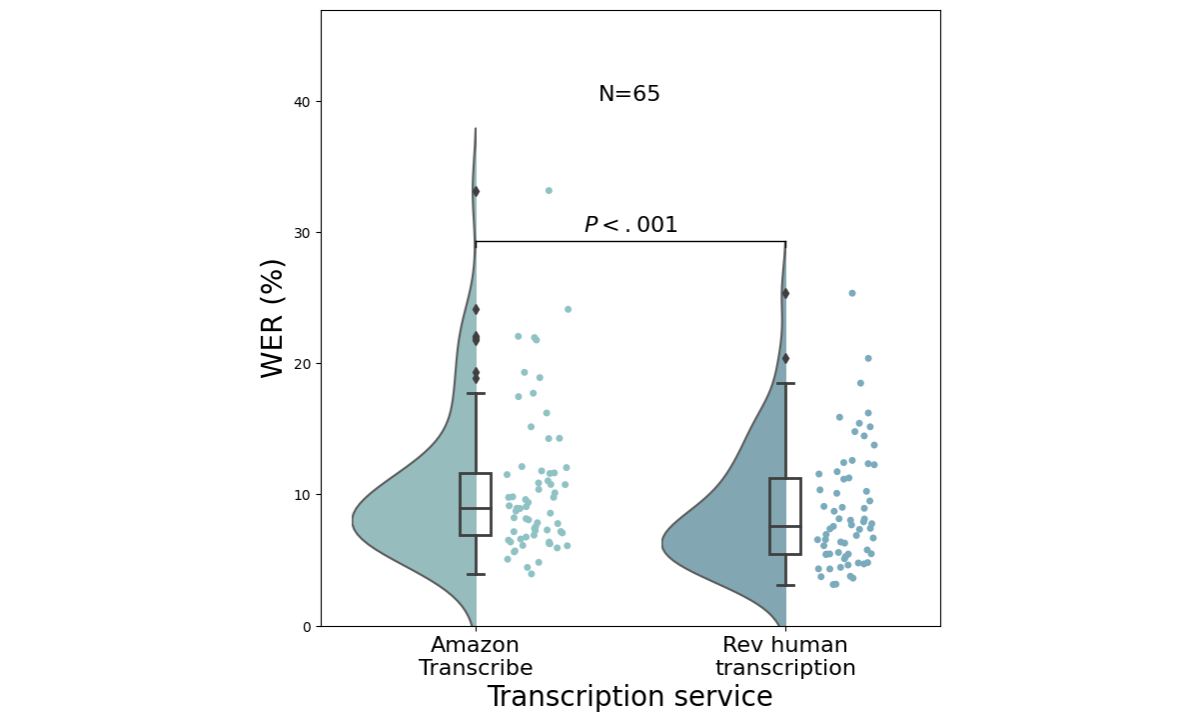
Distributions of the word error rates (WERs) for Amazon Transcribe and Rev human transcription. Dots indicate the actual values of each WER of each of the (N=65) interviewees with shortened audio. Distributions were estimated from the raw values using a kernel density estimate. Box plots indicate the median WER with the 25th and 75th percentiles. *P*<.001 is for a Wilcoxon signed rank test applied between the 2 distributions.

For human transcription provided by Rev, the median WER was at 7.6% (IQR 5.4%-11.3%), and a Wilcoxon paired signed rank test against the Amazon Transcribe indicated a statistically significant difference between these 2 distributions of WERs (*P*<.001). The distributions of Amazon Transcribe’s and Rev’s WER are shown in Figure 5. Table 2 provides the medians, means, and IQRs for all transcription services. However, the errors for each method are not necessarily for the same underlying types of words. It may be incorrect to conclude that either approach is better solely based on aggregate error rate comparisons without deeper analysis of the most common categories or types of erroneous words and without considering their importance for diagnosis.

**Table 2:** Word error rate’s (WER) median (IQR) and mean (SD) as percentages.

Transcription service	WER (%), median (IQR)	WER (%), mean (SD)
Amazon Transcribe	8.9 (6.9-11.6)	10.5 (5.4)
Zoom-Otter AI	19.2 (15.1-24.8)	21.6 (9.1)
Whisper	14.8 (11.1-19.7)	16.7 (7.0)
Rev	7.6 (5.4-11.3)	8.8 (4.5)

### Error Insert and Error Delete by LIWC Category

**Figure 6:**
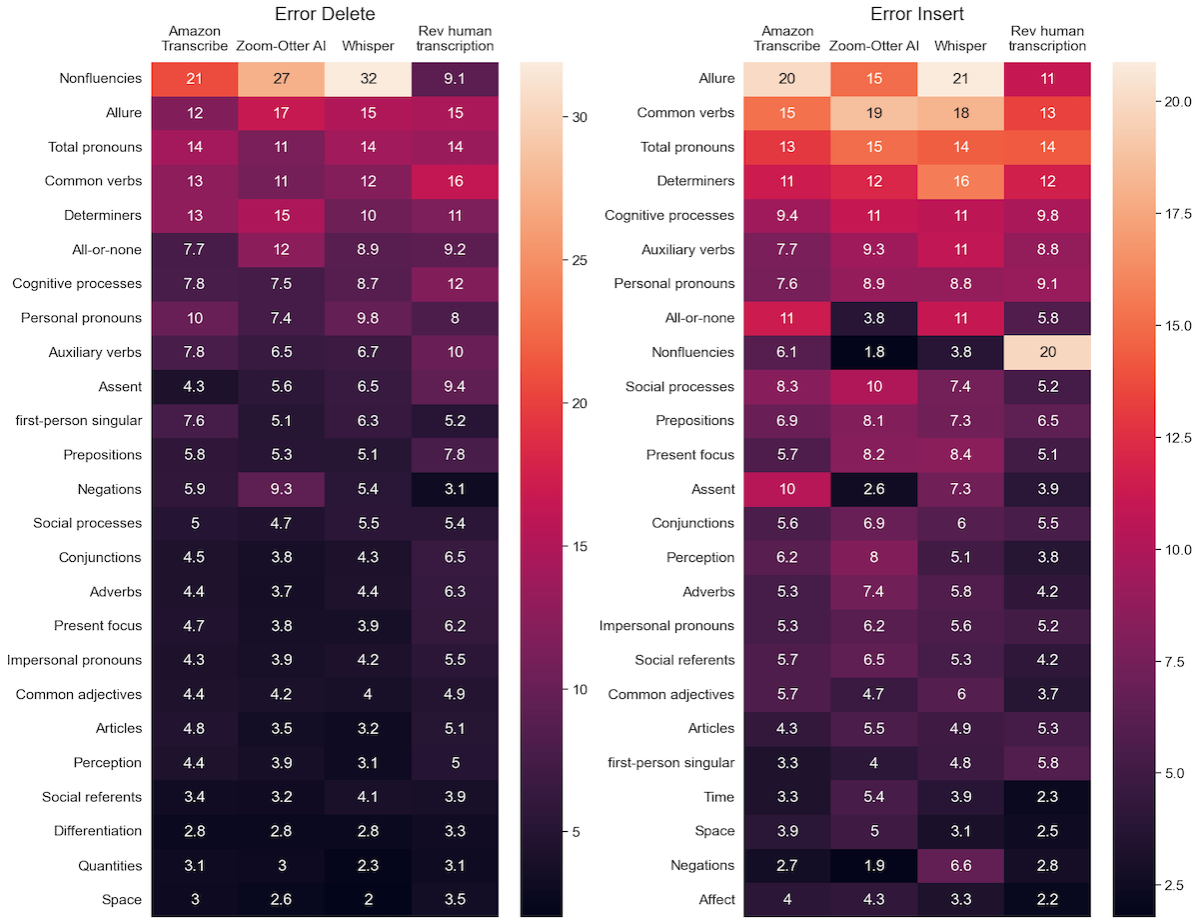
Heat map of top Linguistic Inquiry and Word Count (LIWC) categories (not including the general categories like Conversation, Cognition, and Function) sorted based on average values of all 4 transcriptions for Error Insert (insertion + substitution insertion) as well as Error Delete (deletion and substitution deletion). The numbers represent the percentile contribution of each category to the respective error type, either Error Delete or Error Insert. Major categories (Conversation, Cognition, and Function) have been dropped to accentuate their subcategories (nonfluencies and assent subcategories of Conversation; all-or-none and cognitive processes sub-categories of Cognition; common verbs, auxiliary verbs, determiners, personal pronouns, and total pronouns subcategories of Function).

**Figure 7:**
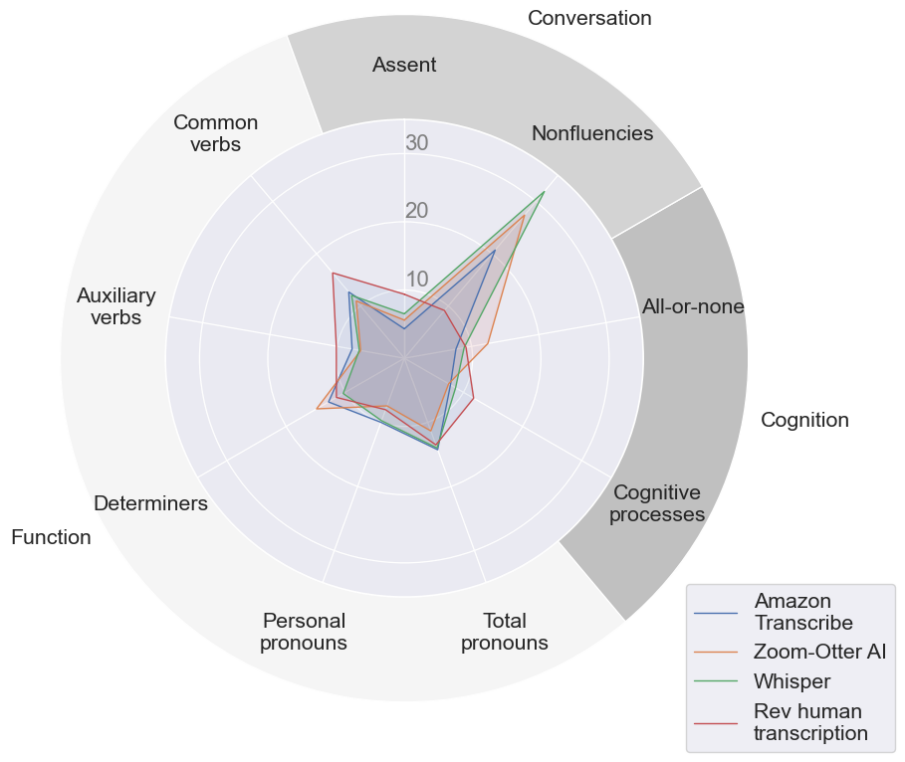
Top Linguistic Inquiry and Word Count (LIWC) categories for Error Delete (deletion and substitution deletion). For each transcription service, these show what percent of Error Delete words are in each category. Different colors represent different transcription services. The outermost labels are the major categories (Conversation, Cognition, and Function); each can be divided into subcategories (nonfluencies and assent for Conversation; all-or-none and cognitive processes for Cognition; common verbs, auxiliary verbs, determiners, personal pronouns, and total pronouns for Function).

**Figure 8:**
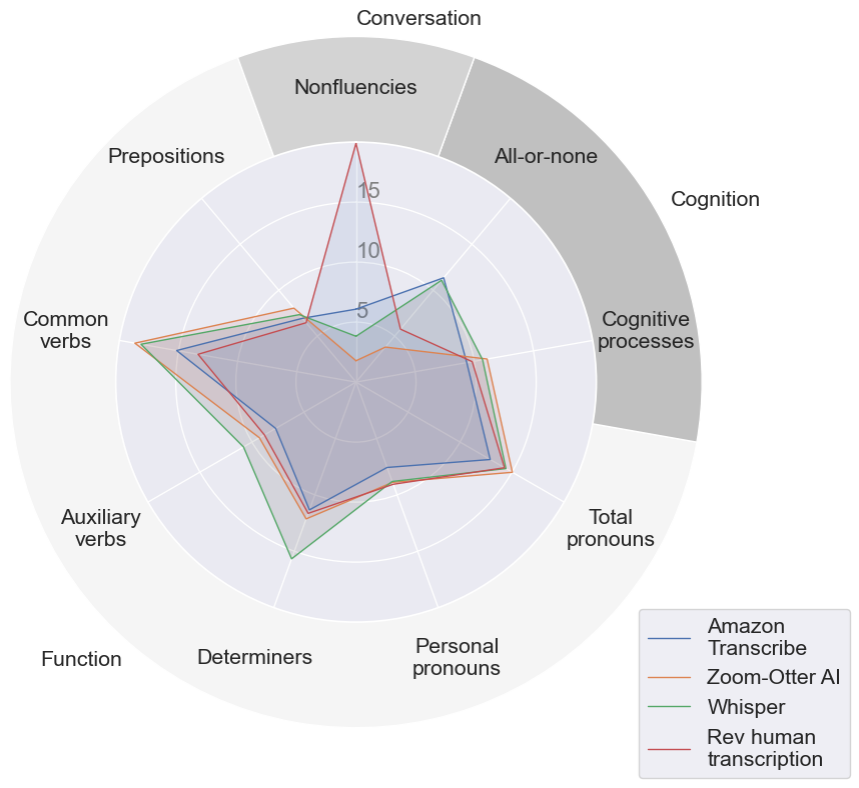
Top Linguistic Inquiry and Word Count (LIWC) categories for Error Insert (insertion + substitution insertion). For each transcription service, these show what percent of Error Insert words are in each category. Assorted colors represent different transcription services. The outermost labels are the major categories (Conversation, Cognition, and Function); each can be divided into subcategories (nonfluencies for Conversation; all-or-none and cognitive processes for Cognition; prepositions, common verbs, auxiliary verbs, determiners, personal pronouns, and total pronouns for Function).

While the WER tallies errors, it fails to distinguish between specific types of errors, such as omissions and insertions. In other words, it does not accurately differentiate between instances where words are mistakenly overrepresented or underrepresented. To further understand the types of errors within these transcription services, the Error Delete and Error Insert percentages were analyzed by LIWC category for all 4 transcription services. There are general overarching LIWC categories, such as “Conversation, Cognition, and Function,” that are composed of subcategories. For example, Conversation includes categories such as assent and nonfluencies [22]. Nonfluencies refer to words such as “oh,” “um,” and “i i,” which are often used in speech [22]. Cognition is a newly added overarching category that is meant to reflect differing ways people think or refer to their thinking, such as through the subcategory of all-or-none thinking [22]. Function words are made up of short, common words such as pronouns, verbs, and determiners [22]. Dropping the abovementioned general overarching categories, the 25 categories with the highest Error Delete and Error Insert based on the average values of all 4 transcriptions are shown through the heat maps in Figure 6. To further visualize these errors, spider plots were created for the top 9 LIWC categories that fell under the overarching categories of Conversation, Cognition, and Function, which were found to have a high percentage of Error Insert and Error Delete. Figure 7 visualizes the error delete by LIWC category, and Figure 8 visualizes the Error Insert by LIWC category.

## Discussion

### Principal Findings

Using a mental health research assessment in an over-the-internet format, we found significant differences in performance, as measured by WER, between services from Amazon Transcribe, Zoom-Otter AI, Whisper, and Rev human transcription. We found no significant differences in transcription errors between the control and MHC groups for Amazon Transcribe, Zoom-Otter AI, and Whisper. Notably, Amazon Transcribe performed significantly better than other tested ASR systems and was very similar to human transcription services, with a marginally higher median transcription WER (8.9%, IQR 6.9%-11.6% vs 7.6%, IQR 5.4%-11.3%).

### WER Performance Across Clinical Categories

To date, there has been a paucity of literature evaluating the performance of ASR across different psychiatric clinical categories. In theory, systematic performance differences could result in discrimination against a particular subset and limit clinical applicability. To alleviate this concern, analysis of the performance of each clinical category is crucial and allows us to detect differences in performance. We found that the services do not seem to discriminate between any particular clinical category, at least in a sample of controls and outpatients (Figure 3). The lack of a statistically significant differences (*P*≥.05) between clinical categories for each transcription service could potentially be explained by a relatively small sample size compared to the large spread of differences. Further research is needed with larger samples; separate analyses of more specific diagnostic categories (individuals with bipolar disorder or schizophrenia-spectrum disorders); and the inclusion of diverse samples encompassing individuals with various cultural, racial, and ethnic backgrounds. In this study, we are focused on comparing the performance of different transcription services using the WER metric and investigating any potential systematic biases present in these errors. While our analysis presented here does not aim to use the transcriptions to differentiate between control and mental health groups, we anticipate in the future that natural language processing methods, and large language models in particular, will be used on the transcribed data to identify mental health status. The performance of these complex models as a function of WER rate in particular word types is unknown, and a statistically insignificant difference may still be amplified by a complex classifier or predictor. Therefore, each of these must be stress-tested as a function of the specific categories of transcription errors.

### WER Performance by Transcription Service

The WERs of 3 automatic services illustrated that Amazon Transcribe outperformed Zoom-Otter AI and Whisper transcriptions. However, when comparing Amazon Transcribe and a human transcription service, Rev performed statistically significantly better despite having a similar median WER. Nevertheless, the WERs were similar to Amazon Transcribe, with a notable difference in nonfluencies (Figures 7 and 8). It is unclear whether this difference is clinically significant, and this is left to future work when we have collected more data. Although these differences were found to be significant, the gap in performance between ASR and human transcription services appears to be narrowing. With overall improvements in ASRs and significantly different service costs compared with human transcription services, ASRs may be the preferred choice if selected wisely.

### Cost and Scalability

Although the Rev human transcription service provided a statistically significantly lower WER (*P*<.01) with a promised delivery time of fewer than 12 hours, it is costly (US $1.50 per min, 50 times more expensive compared with Amazon Transcribe, which is US $0.024 per min) and not as fast as automatic transcriptions (which are almost instantaneous). Also, when scalability is a concern, any human-dependent process can be a rate-limiting step. Moreover, the performance of Amazon Transcribe and human transcription was comparable when looking at both the distributions and the median WER for Amazon at 8.9% (IQR 6.9%-11.6%) versus Rev at 7.6% (IQR 5.4%-11.3%). Our results provide some tentative justification that Amazon Transcribe may serve as a valuable substitute for human transcription, albeit with a few caveats (see the *Limitations and Future Directions* section).

### Errors by LIWC Category

It is important to not only understand the overall WER of these various transcription services but to contextualize the types of words being deleted or inserted in error. As shown through the heat maps and spider plots, the majority of errors fall within 3 overarching LIWC categories: Function, Cognition, and Conversation. Pronouns, specifically first-person and personal pronouns, are often cited as potential predictors of various mental illnesses, most notably depression [23,25,44]. Thus, depending on the population being examined, the use of ASR within LIWC research may provide a misrepresentation of pronoun usage. The same can be seen for words related to cognition, specifically in the all-or-none and cognitive processes categories. There were also a large number of nonfluencies that were deleted in error by the ASR platforms. Nonfluencies have been found to correlate strongly with depression and anxiety in both written and spoken text [45,46]. The nonfluencies category in LIWC is comprised of only 21 words [22], yet it has one of the highest Error Delete percentages for all 3 ASR transcription services. Other LIWC categories commonly noted as potential predictors of mental illness include numerous affect-related categories, such as sadness or negative emotion words [23,24]. However, these affect-related categories were not found to be largely represented in Error Delete or Error Insert within this sample. This could result from this category being less used by participants in these transcriptions or from these types of words being correctly transcribed.

### Limitations and Future Directions

While this study revealed significant differences among ASR transcription services, it is important to acknowledge that 1 of the 3 ASR services (Zoom-Otter AI) used live transcription to produce the transcriptions. This approach could potentially increase the difficulty of the task and lead to reduced accuracy due to limitations in using upcoming parts of speech. However, Amazon Transcribe and Whisper transcribe the audio data in a rolling buffer rather than using the entire recording, and Zoom-Otter AI also has a delay and retrospective correction buffer, which potentially makes the algorithms’ implementations essentially equivalent. We note, however, that pauses and silences longer than 1 second were therefore not removed before transcription by Zoom-Otter AI.

Another potential limitation lies in the fact that the 2 human transcribers had the opportunity to see the agreements and disagreements of the Amazon Transcribe and Zoom-Otter AI outputs before making their corrections. (As noted in the methods, we followed earlier work of Neamatullah et al [39] for deidentification of medical data by combining the strengths of sensitive algorithms and specific humans, which was shown to be highly effective.) This leads to the potential that both human overreaders were “primed” in some manner (and in the same manner) by the transcriptions of the 2 comparative algorithms. However, humans are also primed in some manner by their formative experiences, and there is no evidence to suggest that this is necessarily any more “unbiased” than the algorithms with a human overread. In fact, our Rev human transcription results indicate that humans are very similar to algorithms (on average) but make different mistakes. To identify and remove any residual bias, we would have to have a large (or unknown) number of humans from varying backgrounds and cultures. In other medical data experiments combining human decisions, we have found that the number of individuals required to provide a confident decision or label can be as high as 9 [47]. While identifying the exact number of humans needed to create a near-perfect (or unbiased) transcription is an exciting potential research avenue, it is beyond the scope (and means) of this study.

Finally, we note that this study’s findings are limited by the relatively small sample size and the demographics skewing toward White, highly educated females. It is also important to note that there was relatively little background noise in our recordings, and some individuals, particularly those with fewer resources, may not be able to find quiet locations to talk over video. However, innovations in background noise cancellation, particularly other voices, have improved enormously over the last few years and somewhat mitigate this issue.

Given the increasing use of ASR transcription services in clinical settings, independent evaluations of WERs are crucial to ensuring these services are accurate for specific contexts. It is currently unclear whether a given WER would alter the clinical decision-making process or outcomes for a particular patient or for a given algorithm that uses the transcribed text as input. Future research should further examine the types of errors these transcription services are getting and whether the contents of those words are clinically impactful. Even seemingly minor errors have the potential to cause clinically significant errors in diagnoses or treatment recommendations, for example, if ASRs are not thoroughly tested in the context in which they are to be used. Moreover, biases in ASR algorithms (eg, those that have been trained on “standard English”) may further exacerbate diagnostic disparities or lead clinicians to select improper treatments, particularly in the case of underrepresented minority groups, women, nonnative English speakers, and individuals from low socioeconomic or low literacy groups. It will be important to incorporate measures for such subgroups and implement methods to mitigate these disparities at both the algorithmic and user-implementation levels. Such analysis will require a much larger corpus of data. These issues will be the subject of subsequent publications on our corpus as we continue to increase the size and diversity of our population.

### Conclusions

The gap in performance between ASR and human transcription services continues to narrow, and our results appear to indicate that they are close to being equivalent. This is consistent with the trend in the literature where, depending on the context, WER has dropped from around 30% in the early 2000s [5] to 10% to 15% in the 2010s [6] to under 10% in recent years [7]. With overall improvements in ASRs and significantly lower service costs (around 50 times less expensive) compared with human transcription services, ASRs are increasingly likely to be the preferred choice for medical transcription. However, further research needs to evaluate various clinical populations with larger, more diverse sample sizes to determine whether these errors impact the analysis and usability of these ASR transcriptions in applied settings. Of course, human transcription services should be subject to the same analysis, as they also have the potential to generate the same biases and errors.

## Abbreviations

ASR: automatic speech recognition
EHR: electronic health record
HIPAA: Health Insurance Portability and Accountability Act
LIWC: Linguistic Inquiry and Word Count
MHC: mental health condition
MINI: Mini-International Neuropsychiatric Interview
TAT: thematic apperception test
WER: word error rate
